# Widely Used Pesticides with Previously Unknown Endocrine Activity Revealed as *in Vitro* Antiandrogens

**DOI:** 10.1289/ehp.1002895

**Published:** 2011-02-10

**Authors:** Frances Orton, Erika Rosivatz, Martin Scholze, Andreas Kortenkamp

**Affiliations:** Centre for Toxicology, School of Pharmacy, University of London, London, United Kingdom

**Keywords:** antiandrogen, AR-Lux, biomonitoring, endocrine disruption, fungicide

## Abstract

**Background:**

Evidence suggests that there is widespread decline in male reproductive health and that antiandrogenic pollutants may play a significant role. There is also a clear disparity between pesticide exposure and data on endocrine disruption, with most of the published literature focused on pesticides that are no longer registered for use in developed countries.

**Objective:**

We used estimated human exposure data to select pesticides to test for antiandrogenic activity, focusing on highest use pesticides.

**Methods:**

We used European databases to select 134 candidate pesticides based on highest exposure, followed by a filtering step according to known or predicted receptor-mediated antiandrogenic potency, based on a previously published quantitative structure–activity relationship (QSAR) model. In total, 37 pesticides were tested for *in vitro* androgen receptor (AR) antagonism. Of these, 14 were previously reported to be AR antagonists (“active”), 4 were predicted AR antagonists using the QSAR, 6 were predicted to not be AR antagonists (“inactive”), and 13 had unknown activity, which were “out of domain” and therefore could not be classified with the QSAR (“unknown”).

**Results:**

All 14 pesticides with previous evidence of AR antagonism were confirmed as antiandrogenic in our assay, and 9 previously untested pesticides were identified as antiandrogenic (dimethomorph, fenhexamid, quinoxyfen, cyprodinil, λ-cyhalothrin, pyrimethanil, fludioxonil, azinphos-methyl, pirimiphos-methyl). In addition, we classified 7 compounds as androgenic.

**Conclusions:**

Due to estimated antiandrogenic potency, current use, estimated exposure, and lack of previous data, we strongly recommend that dimethomorph, fludioxonil, fenhexamid, imazalil, *ortho*-phenylphenol, and pirimiphos-methyl be tested for antiandrogenic effects *in vivo*. The lack of human biomonitoring data for environmentally relevant pesticides presents a barrier to current risk assessment of pesticides on humans.

Evidence suggests that prenatal and early-life exposure to pesticides may be causative factors in a variety of human disorders. For example, a meta-analysis by Wigle et al. (2009) showed that maternally exposed offspring have increased risk of childhood leukemia [odds ratio = 2.64; 95% confidence interval (CI), 1.4–5].

There are also indications that reproductive abnormalities, expressed as cryptorchidism, hypospadias, and decreased penile length, may be linked to pesticide exposure, most strikingly in maternally exposed boys (Andersen et al. 2008; Damgaard et al. 2006; Rocheleau et al. 2009). This is significant because male fertility is thought to be declining in many countries (Andersson et al. 2008), and perinatal hypospadias/cryptorchidism are risk factors for reduced sperm quality and testicular cancer in adulthood (Skakkebaek et al. 2001). Banned persistent organochlorines [*p*,*p*′-DDT (1,1,1-trichloro-2-[*o*-chlorophenyl]-2,2-[*p*-chlorophenyl]ethane), *p*,*p*′-DDE (*p*,*p*′-1,1-*bis*-(4-chlorophenyl)-2,2-dichloroethene), β-hexachlorocyclohexane, hexachlorobenzene, α-endosulfan, *cis*-heptachloroepoxide, oxychlordane, dieldrin] were detected in all samples of breast milk in a case–control study of mothers in Denmark and Finland. Also, levels were significantly higher in samples from mothers of sons with cryptorchidism than in samples from matched controls (1997–2001; Damgaard et al. 2006). Female Danish greenhouse workers exposed to current-use pesticides were more likely to give birth to a son with cryptorchidism than were a random sample of mothers from the Copenhagen area (6.2% and 1.9%). Furthermore, sons of mothers who directly handled treated plants or were engaged in spraying pesticides had significantly smaller penises than did sons of mothers who had noncontact roles in the greenhouse industry (Andersen et al. 2008). Last, in a recent meta-analysis of studies from the United States and Europe, Rocheleau et al. (2009) reported that maternal occupational exposure to pesticides was associated with a 36% increased risk of hypospadias relative to the risk in mothers without exposure (risk ratio = 1.36; 95% CI, 1.04–1.77). The risk of developing cryptorchidism (Pierik et al. 2004) and hypospadias (Brouwers et al. 2007) was also associated with paternal exposures to pesticides, mainly in greenhouses for the production of vegetables and flowers.

The term “testicular dysgenesis syndrome” (TDS) has been proposed to explain the interrelated nature of these abnormalities (Skakkebaek et al. 2001). It is conceivable that estrogenic and/or antiandrogenic contaminants play a role in TDS. Experimental studies with rats have shown that maternal exposure to flutamide (a pharmaceutical antiandrogen) affects androgen-dependent developmental outcomes such as anogenital distance and nipple retention (McIntyre et al. 2001). However, ethinylestradiol has not been shown to affect these end points (Howdeshell et al. 2008). Furthermore, hormone receptor screening *in vitro* suggests a preponderance of antiandrogenic activity compared with estrogenic activity in nonorganochlorine (current-use) pesticides. For example, Kojima et al. (2004) screened 161 pesticides and reported that 52 were antiandrogenic, whereas only 29 were estrogenic, and Orton et al. (2009) reported that 6 of 12 pesticides screened were antiandrogenic and none were estrogenic. There is a good correlation between androgen receptor (AR) antagonist properties and *in vivo* antiandrogenic effects, and there is also good evidence that androgen-sensitive end points are demasculinized in male rats when exposed *in utero* to a wide range of pesticides. Antiandrogenic effects both *in vitro* and via maternal exposure *in vivo* have been reported in response to the herbicide linuron (Gray et al. 1999; Lambright et al. 2000); the fungicides prochloraz (Vinggaard et al. 2005), procymidone (Ostby et al. 1999), tebuconazole (Taxvig et al. 2007), and vinclozolin (Anway et al. 2006; Uzumcu et al. 2004); the organochlorine insecticides DDE (Gray et al. 1999) and endosulfan (Sinha et al. 2001); the organophosphate dimethoate (Verma and Mohanty 2009); and the pyrethroid insecticide deltamethrin (Andrade et al. 2002). However, with the exception of linuron, dimethoate, deltamethrin, and tebuconazole, the pesticides listed above have not been authorized for use in Europe during the past 5 years, which should result in lower occupational, residential, and dietary exposures. Endocrine-relevant data on current use pesticides is minimal—and in some cases completely absent—with most of the published literature focused on pesticides that are no longer registered for use.

Therefore, the aim of this study was to test the antiandrogenic activity of currently used pesticides, with a view to informing future studies to determine their likely role in causing TDS. We selected compounds for testing based on evidence of human exposure (dietary intake data for Europe) and predicted AR antagonism according to the quantitative structure–activity relationship (QSAR) model developed by Vinggaard et al. (2008). Compounds predicted to be AR antagonists and compounds with high exposure scores were analyzed for AR antagonist properties using the MDA-kb2 assay (Ermler et al. 2010; Wilson et al. 2002). In addition, we used the yeast antiandrogen screen (YAS) to further test a subset of pesticides that were newly identified as AR antagonists or that had MDA-kb2 assay results that were discordant with QSAR predictions.

## Materials and Methods

### Test compound selection

Pesticides were selected using a combination of exposure scores and data about receptor-mediated antiandrogenic activity [see Supplemental Material, Figure 1 (doi:10.1289/ehp.1002895)]. First, we identified 134 pesticides with data suggesting relevant human exposures, including 58 pesticides identified at the highest concentrations and most frequently in European foods (European Commission 2008); 30 additional pesticides with relatively high daily dietary intakes (> 0.0004 μg/kg/day) identified by the FAO/WHO (Food and Agriculture Organization of the United Nations/World Health Organization) Joint Meeting on Pesticide Residues (JMPR) (FAO/WHO 2011); 44 additional pesticides identified in > 0.4% of fruits and vegetables during routine testing [European Food Safety Authority (EFSA) 2009]; and *o*,*p*′- and *p*,*p*′*-*DDE, which we included because of known adipose tissue levels (Fernández et al. 2004). Each pesticide was assigned four scores, with each ranging from 1 to 10: *a*) maximum food residue level (European Commission 2008); *b*) estimated daily dietary intake (FAO/WHO 2011); *c*) frequency of detection in fruits and vegetables (EFSA 2009), with a score of 5 assigned when data were not available; and *d*) a score according to the number of times pesticides were listed as one of the top 10 pesticides identified in fruits and cereals in Europe (a frequency score), with a score of 0 assigned if they were never listed (European Commission 2008). The four scores were summed to generate a “total exposure score,” with a maximum possible score of 40 (see Supplemental Material, Table 1).

The second stage of compound selection for testing was an assessment of *in vitro* evidence of AR interaction in the available literature (Andersen et al. 2002; Bauer et al. 2002; Kojima et al. 2004; Okubo et al. 2004; Orton et al. 2009; Vinggaard et al. 2008). Compounds previously shown not to be AR antagonists *in vitro* (*n* = 43) were removed from the list, which reduced the number of candidate pesticides from 134 to 91. Compounds previously reported to be AR antagonists (*n* = 27) were removed if the ratio of their total exposure score to their published IC_20_ [concentration that inhibits the androgenicity of DHT by 20%; total exposure score/published IC_20_ = “environmental relevance ratio” (ERR)] was < 3 (ERR was recalculated using our experimental data after the selection process). This left 14 previously reported AR antagonists for testing by the MDA-kb2 assay. For pesticides without published data (*n* = 64), AR antagonist activity was predicted using the QSAR developed by Vinggaard et al. (2008). These pesticides were tested using the MDA-kb2 assays if they were predicted to have AR antagonist activity (*n* = 4) or if they had high exposure scores (> 8) regardless of their QSAR status, including 6 pesticides that were predicted not to have AR antagonist activity and 13 pesticides that could not be predicted because they were out of the domain of the QSAR model. In total, 37 compounds were selected for testing in the MDA-kb2 assay. Finally, 8 pesticides that were newly described as highly active antiandrogens in the MDA-kb2 assay and 4 pesticides for which the QSAR prediction differed from the experimental result (including 1 out of the model domain) were subjected to further testing using the YAS (*n* = 14). For a summary of the selection process, see Supplemental Material, Figure 1 (doi:10.1289/ehp.1002895).

### Chemicals

Dihydrotestosterone (DHT; > 97% purity) was purchased from Steraloids Ltd. (Croydon, Surrey, UK); novaluron, dimethomorph, *p*,*p*′-DDE, methiocarb, and indoxacarb were purchased from Greyhound Chromatography and Allied Chemicals (all > 98.7% pure; Birkenhead, Merseyside, UK); and all other pesticides (all > 97% pure) were purchased from Sigma Aldrich (Poole, Dorset, UK). Ethanol (> 99.7% purity) was obtained from VWR International Ltd. (Leicestershire, UK). All test compounds were dissolved in ethanol to make stock solutions to be used in the assays.

### MDA-kb2 assay

MDA-kb2 cells are human breast cancer cells stably transfected with a firefly luciferase reporter gene that is driven by an androgen-response element–containing promoter (Wilson et al. 2002). Details of the modified assay were published previously (Ermler et al. 2010). Briefly, cells were seeded at a concentration of 1 × 10^5^ cells/mL in phenol red–free Leibowitz-15 medium (Invitrogen Ltd., Paisley, UK) containing 10% (charcoal-stripped) fetal calf serum (Invitrogen Ltd.) in white luminometer plates and allowed to attach for 24 hr. Cells were then exposed to eight serial dilutions of selected pesticides with or without DHT (0.25 nM). After 24 hr, luciferase activity was determined with SteadyGlo assay reagent (Promega UK Ltd., Southampton, Hampshire, UK) and measured in a plate reader (FLUOstar Optima, BMG Labtech GmbH, Offenburg, Germany). The following controls were run on each plate: media, ethanol, DHT coexposure (0.25 nM), DHT serial dilutions (0.002–10 nM), and flutamide (0.013–8 μM) or procymidone (0.005–3.2 μM) serial dilutions. All concentrations were tested in duplicate over two plates, and each pesticide was measured at least twice in separate experiments. For comparative purposes, luminescence was normalized to DHT alone at coexposure concentration (maximum response, 100%) and solvent-only (ethanol) controls (minimum response, 0%). Initially, flutamide was used as the internal quality control for antiandrogenicity; however, because of overlap of toxic effects on the cells with antiandrogenic activity, it was replaced by procymidone, which is more potent [IC_50_ (50% concentration that inhibits): flutamide, 1.56 μM; procymidone, 0.53 μM] but nontoxic to MDA-kb2 cells in the concentration range associated with receptor antagonism. Pesticides were initially tested over a concentration range of 0.64 nM–50 μM (5× dilutions) as a range-finding exercise. Subsequently, the concentration ranges were modified to reflect the potency and toxicity of each individual compound. Because cytotoxic effects could not be distinguished from antiandrogenic effects in the coexposed treatments, any readings of the pesticide statistically significantly below the mean ethanol control level (0%) were considered toxic to MDA-kb2 cells, and the corresponding coexposure data were not classified as antiandrogenic. Sixty percent of the pesticides were repeat tested using the same product but with new stock solutions and by a different experimenter.

### YAS

The methods for the YAS have been described previously (Sohoni and Sumpter 1998). Briefly, stimulation of the transfected AR causes a color change in the media, which is measured by absorbance at 540 nm (Labsystems Multiskan Multisoft, Vienna, VA, USA). Plates were also measured at 620 nm to measure cell growth (turbidity) to check for any cytotoxic effects that may have occurred. Pesticides were coincubated with DHT (6.4 nM). Controls run in each experiment were ethanol, DHT serial dilutions (0.0026–100 nM), and flutamide serial dilutions (0.19–100 μM). The pesticide concentration range varied according to potency observed in MDA-kb2 assay but was between 0.016 and 750 μM for all test compounds. Incubation time was 53 hr at 28°C. Where turbidity readings were significantly depressed, toxicity was indicated and the effect could not be considered antiandrogenic; therefore, these dilutions were removed from analysis. Pesticide serial dilutions were tested in duplicate over two plates and were tested in two separate experiments.

### Statistics

To analyze antiandrogenic action, raw luminescence readings were normalized on a plate-by-plate basis to the means of the positive DHT controls (*n* = 8) and the solvent controls (*n* = 8) (Ermler et al. 2010). We pooled all data from the same test compound and conducted statistical concentration–response regression analyses using the best-fit approach (Scholze et al. 2001). Specifically, a variety of nonlinear regression models were fitted independently to the same data set, and the best-fitting model was selected using a statistical goodness-of-fit criterion. Concentration–response data from different researchers were first analyzed one by one using regression models, and differences in regression analyses due to data from different researchers were judged as statistically significant when the 95% CIs of the regression curves did not overlap. Such statistical differences between researchers were not observed, and thus data were pooled for final analysis. Luminescence readings from pesticides tested in the absence of DHT were divided by the mean of the solvent controls from the same plate and analyzed for negative and positive trends (suggestive of cytotoxic or androgenic action, respectively) by nonparametric contrast tests (Neuhaeuser et al. 2000). Data considered to be statistically significant at *p* < 0.05 were analyzed using the best-fit approach as described above. All statistical analysis was performed using SAS statistical software (SAS Institute Inc., Cary, NC, USA). From the best-fitting model, we derived inhibitory concentrations for antiandrogenicity and effect concentrations for cytotoxicity.

## Results

**Figure 1:**
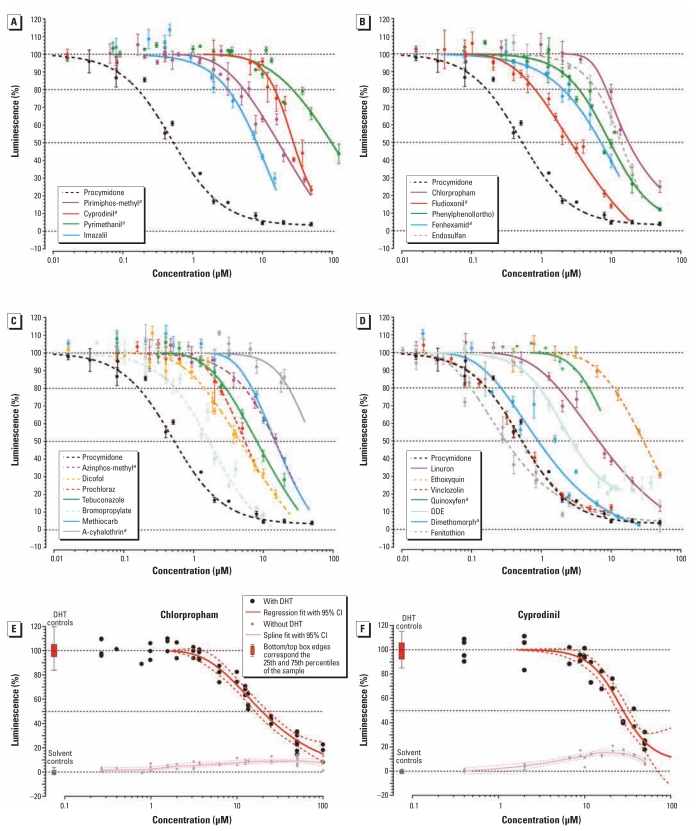
Results of the MDA-kb2 assay showing regression curves for antiandrogenic pesticides (*A*–*D*) and stimulatory activity for chlorpropham (*E*) and cyprodinil (*F*). Values for luminescence were normalized to those of controls. In *A–D*, compounds are grouped by exposure scores (see Table 1), from highest (*A*) to lowest (*D*), with procymidone shown in each as a point of reference. Regression lines end at the toxic threshold. Dashed lines indicate pesticides with lapsed registration, and solid lines indicate pesticides with current registration; data shown are mean ± SE. Data for chloropham (*E*) and cyprodinil (*F*) demonstrate overlap of AR antagonism (black data points and curves) with receptor agonism (gray curves). *^a^*Newly described antiandrogens.

We derived the within-plate variation from readings of the positive DHT controls as a coefficient of variation (CV), with 95% of all CVs falling between 2.1% and 12.9% (mean, 6.5%). Of the 37 tested compounds, 24 pesticides were antiandrogenic in the MDA-kb2 assay, 9 of which are newly described (Table 1, Figure 1). The most potent *in vitro* AR antagonist was fenitrothion (IC_20_ = 0.098 μM), and the least potent was pyrimethanil (IC_20_ = 27.2 μM). All 14 compounds previously reported in the literature as antiandrogenic were confirmed using our test system. Two of 4 previously untested pesticides that were predicted to be AR antagonists in the QSAR were positive in the MDA-kb2 assay, and 3 of 13 pesticides that could not be predicted using the QSAR (i.e., they were out of the model domain) were also antiandrogenic. Five of 6 pesticides predicted to be inactive based on the QSAR were AR antagonists in the MDA-kb2 assay, but 3 were out of the QSAR prediction range because they were antiandrogenic at a concentration higher than the exclusion criterion of the QSAR (limit of detection, IC_25_ ≤ 10 μM; IC_20_: cyprodinil, 15.1 μM; pyrimethanil, 27.2 μM; cyhalothrin, 23.1 μM).

All 14 pesticides tested using the YAS were antiandrogenic, including two that lacked activity in the MDA-kb2 assay [tolylfluanid (out of domain of QSAR) and bifenthrin (predicted active in QSAR)] (Table 1).

Twenty-two of the 37 pesticides analyzed in the MDA-kb2 assay were cytotoxic. The concentrations required to elicit cytotoxicity were between 2.1 times (quinoxyfen) and 50 times (bromopropylate) higher than the concentrations associated with antiandrogenicity [based on the ratio of EC_20_ (concentration that produces a 20% effect) for cytotoxicity and IC_20_ for antiandrogenicity]. Seven of the chemicals analyzed in the MDA-kb2 assay showed AR agonist activity when tested in the absence of DHT coexposure, including two (cyprodinil and chlorpropham) with androgenic activity occurring at lower concentrations than antiandrogenic activity (Table 1, Figure 1). Four of 14 pesticides were cytotoxic in the YAS assay (cyprodinil, pyrimethanil, tolylfluanid, and difenoconazole), whereas we observed no AR agonism in this assay (Table 1).

## Discussion

Our results indicate that systematic testing for antiandrogenic activity of currently used pesticides is urgently required. For example, 20 of the 50 pesticides with the highest exposure scores were antiandrogenic in at least one assay, including 8 that have not been identified as antiandrogens previously [see Supplemental Material, Figure 2 (doi:10.1289/ehp.1002895)]. In previous *in vitro* screenings of current-use pesticides, proportions of antiandrogenic pesticides were broadly similar [32% (52 of 161), Kojima et al. 2004; 50% (6 of 12), Orton et al. 2009; 62% (38 of 61), Vinggaard et al. 2008], further supporting the possibility that a large fraction of untested pesticides may be antiandrogenic. In contrast, estrogenic activity appears to be less common in current-use pesticides [18% (29 of 161), Kojima et al. 2004; 0% (0 of 100), Nishihara et al. 2000; 0% (0 of 12), Orton et al. 2009]. Some discrepancy between our data and published data exists; for example, pirimiphos-methyl was previously reported to have no antiandrogenic activity (Kojima et al. 2004), and chlorpropham has been reported to have no activity (Kojima et al. 2004) and to be antiandrogenic (Orton et al. 2009). These differences are most likely due to differences among the assay systems used. We also observed differences between findings based on the MDA-kb2 assay and the YAS assay. However, IC_20_ values based on the two assays never deviated by more than one order of magnitude, with the exception of two pesticides (tolylfluanid, bifenthrin) that were cytotoxic in the MDA-kb2 assay, and dimethomorph, for which we observed a large divergence in AR antagonist activity (IC_20_: MDA-kb2, 0.263 μM; YAS, 38.5 μM).

We did not design our study to evaluate the QSAR by Vinggaard et al. (2008), and the number of chemicals falling within the applicability domain of the model was low; however, we note that several pesticides with antiandrogenic activity *in vitro* were not predicted by the QSAR, in part because some of the compounds were less potent than the prediction domain of the QSAR, which classifies chemicals with an IC_25_ > 10 μM as devoid of antiandrogenicity. The large percentage of pesticides for which the QSAR was not able to provide predictions (45 of 64) suggests that extending the applicability domain would increase the usefulness of the model.

The ranking according to our exposure scoring system was similar to the listed “adjusted theoretical maximum dietary intake” of pesticides (58% concordance among the top 40 compounds) previously reported by Menard et al. (2008), which is based on actual French consumption data and maximum residue levels. Consequently, the ERR was similar, using either our exposure scores or the adjusted theoretical dietary intake published by Menard et al. (2008) [see Supplemental Material, Table 2 (doi:10.1289/ehp.1002895)]. Both our exposure data and those used by Menard et al. (2008) were sourced from before 2008 (except JMPR reports from 2008 and 2009) and therefore may not be fully representative of current exposures. Indeed, from 2005 through 2010, the authorizations for use granted by European Union authorities expired for 12 of the tested pesticides, including several *in vitro* AR antagonists (procymidone, prochloraz, vinclozolin, ethoxyquin, endosulfan, azinphos-methyl, bromopropylate, dicofol, and fenitrothion) and 3 without evidence of antiandrogenic activity (bifenthrin, propargite, and profenofos). Thus, exposure to some of the tested compounds should decrease, whereas exposure to replacement products may increase. For example, a pesticide formulation called Switch, which contains cyprodinil and fludioxonil (both of which were antiandrogenic in our test system), was recommended as a replacement for the vinclozolin formulation Ronilan (Shah et al. 2002).

To our knowledge, except for two reports to date (Heudorf and Angerer 2001; Saieva et al. 2004), there is a complete absence of published human biomonitoring data for pesticides in Europe, and therefore, it is impossible to predict how the levels eliciting an effect *in vitro* may correspond to human internal concentrations. Similarly, although the National Health and Nutrition Examination Survey (NHANES) in the United States incorporates human biomonitoring of pesticides, exposure concentrations in human target tissues are very poorly understood, because of the almost complete lack of toxicokinetic data, short half-lives of current use pesticides, unspecific urinary metabolites, and unknown metabolic pathways (see Barr 2008). Pesticides with relatively large ERRs, including dimethomorph (expiration of European Union authorization, September 2017), fludioxonil (October 2018), fenhexamid (May 2011), imazalil (December 2011), linuron (December 2013), *ortho*-phenylphenol (December 2019), tebuconazole (August 2019), and pirimiphos-methyl (September 2017), may be important antiandrogenic pollutants at present and in the future (Table 1). Linuron and tebuconazole are known *in vivo* antiandrogens (Lambright et al. 2000; Taxvig et al. 2007); however, data on the other pesticides are much more limited. This is especially true of dimethomorph, fludioxonil, and fenhexamid, for which we were unable to identify previous publications regarding endocrine disruption. These compounds are newly formulated fungicides (dimethomorph, 2007; fludioxonil, 2008; fenhexamid, 2001), which are stable on food commodities (> 70% of the parent compound) and remain unchanged on the commodity when reaching the consumer (EFSA 2007, 2010a, 2010b). Dimethomorph and fenhexamid belong to the fungicide group of sterol biosynthesis inhibitors (Leroux 2004), as do the *in vivo* antiandrogenic conazoles (e.g., Taxvig et al. 2007) and imidazoles (Vinggaard et al. 2005). A study of the sterol biosynthesis inhibitors imazalil, propiconazole, triadimefon, triadimenol, and prochloraz indicated that all inhibited aromatase in human placental microsomes (Vinggaard et al. 2000), but to our knowledge, effects of dimethomorph and fenhexamid on steroidogenesis in mammalian cells have not been assessed. Imazalil and the *in vivo* antiandrogen prochloraz (Vinggaard et al. 2005) are both classified as imidazole fungicides, and *in vitro* potency estimates for the two compounds were similar (IC_20_: imazalil, 3.23 μM; prochloraz, 2.39 μM), but the possible effects of imazalil *in vivo* have not been evaluated. Therefore, it is our view that dimethomorph, fludioxonil, fenhexamid, and imazalil should be tested *in vivo* as a matter of urgency. Another relevant pesticide may also be *ortho*-phenylphenol, which is used as a fungicide in agriculture and as a wood preservative, and also has a wide variety of industrial applications (e.g., preservation of glues, plastic additives in flame retardants, disinfectant in hospitals) (LANXESS Corp. 2010). In our exposure ranking system, it ranked 12th out of 37 test compounds (Table 1). Considering that *ortho*-phenylphenol was highly ranked by exposure and that nonagricultural sources were absent from our exposure scores, it is not surprising that it was detected in all human urine samples tested in two studies [mean concentration, 2.9 nM, *n* = 30 samples (Ye et al. 2005); 35.2 nM, *n* = 22 samples (Bartels et al. 1997)], 85% of breast milk samples [mean concentration, 10.6 nM, *n* = 20 samples (Ye et al. 2006)], and 30% of amniotic fluid samples [mean concentration, 0.76 nM, *n* = 20 samples (Bradman et al. 2003)] in the United States. *ortho*-Phenylphenol was previously identified as a receptor-mediated antiandrogen (Kojima et al. 2004), but no data are available on its possible effects *in vivo*. Pirimiphos-methyl is an organothiophosphate insecticide that is stable on stored grain (< 24 weeks, 70% unchanged parent compound; EFSA 2005). There are also indications that it may be antiandrogenic *in vivo* because maternal and postnatal exposure of rats to 12 mg/kg body weight/day caused testicular tubular atrophy (EFSA 2005). In addition, treatment of adult male rats for 90 days resulted in decreased sperm density and mobility (125 mg/kg body weight/day), testicular atrophy (lowest observed adverse effect level, 41.67 mg/kg body weight/day), and decreased fertility (125 mg/kg body weight/day) (Ngoula et al. 2007). There is insufficient evidence to assess the risk of tested pesticides to human health because of a lack of data. However, to our knowledge, all of the pesticides (with the possible exception of fenitrothion; Okahashi et al. 2005; Turner et al. 2002) identified as *in vitro* AR antagonists in our study have also been reported to have antiandrogenic effects *in vivo* in animal models (Anway et al. 2006; Gray et al. 1999; Lambright et al. 2000; McIntyre et al. 2002; Ostby et al. 1999; Sinha et al. 2001; Taxvig et al. 2007; Uzumcu et al. 2004; Vinggaard et al. 2005). We also identified 7 compounds that appeared to be androgenic because they stimulated activity in the absence of DHT. The mechanism of action for this response is not well characterized; however, it has been previously detected in this assay (Tamura et al. 2006; Wilson et al. 2002) and was proposed to be due to conformational change of the ligand-binding pocket in such a way that simultaneous androgenic and antiandrogenic activities were possible (Tamura et al. 2006). We are unable to confirm or reject these data; however, preliminary data from our laboratory suggests that the stimulatory response is neither via stimulation of the receptor, because we have not observed evidence of androgenic effects in the YAS for any compounds, nor due to cell proliferation, as evidenced by transient transfection of cells with a nonandrogenic responsive element. Cyprodinil and chlorpropham were more potent AR agonists (EC_20_ = 1.91 and 2.67, respectively) than antagonists (IC_20_ = 15.1 and 7.66, respectively) in the MDA-kb2 assay.

## Conclusions

In addition to identifying new candidate antiandrogens, our findings highlight important data gaps that prevent accurate assessment of male reproductive health risks from pesticides. The most important of these are the absence of *in vivo* studies and human biomonitoring data for environmentally relevant pesticides. In addition, fungicides typically had high exposure scores and were thus well represented in the testing set, presumably because they are often applied just before or after harvest to food commodities. They are typically applied as mixtures in order to increase effectiveness and prevent development of resistant strains (Fungicide Resistance Action Committee 2010), and therefore, human exposure to mixtures of these *in vitro* antiandrogens may be considerable. The contribution of pesticides to declining male reproductive health requires further investigation, particularly to clarify the relationship between effective concentrations *in vivo* and exposure.

## Figures and Tables

**Table 1:** Receptor-mediated antiandrogenic activity and cytotoxicity in the MDA-kb2 and YAS assays.

Compound	Expiration^a^	Score^b^	QSAR prediction	Antiandrogen IC_20_ (μM)		Cytotoxic EC_20_ (μM)		Androgen^c^ EC_20_ (μM) MDA-kb2	ERR
				MDA-kb2	YAS	MDA-kb2	YAS		
Fungicides									
Cyprodinil^d^	Apr 2017	33	Inactive	15.1	1.34	> 50	27.8	1.91	2.2
Procymidone^e^	Jun 2008	33	AA	0.163	0.956	> 50	> 160	Neg	202.5
Imazalil	Dec 2011	32	AA	3.23	—	19.0	—	Neg	9.9
Pyrimethanil^d^	May 2017	28	Inactive	27.2	9.15	> 125	167	27.8	1.0
Fludioxonil^d^	Oct 2018	25	OD	0.801	0.730	28.5	> 160	Neg	31.2
Azoxystrobin	Dec 2011	24	Inactive	Neg	—	2.9	—	Neg	NA
Fenhexamid^d^	May 2011	24	Active	2.02	—	21.6	—	Neg	11.9
Tolylfluanid	Sep 2016	24	OD	Neg	0.234	8.09	1.14	Neg	NA
*O*-Phenylphenol	Dec 2019	21	AA	3.43	—	> 50	—	Neg	6.1
Prochloraz^e^	Dec 2010	18	AA	2.39	—	12.5	—	Neg	7.5
Pyraclostrobin	May 2014	17	OD	Neg	—	0.089	—	Neg	NA
Mandipropamid	Jul 2011	18	OD	Neg	—	8.92	—	Neg	NA
Tebuconazole	Aug 2019	16	AA	2.89	—	38.9	—	Neg	5.5
Difenoconazole	Dec 2018	13	Active	Neg	Neg	2.91	0.109	Neg	NA
Vinclozolin^e^	Jan 2007	13	AA	0.163	—	> 50	—	2.9	79.8
Dimethomorph^d^	Sep 2017	12	Active	0.263	38.5	> 25	> 50	Neg	45.6
Quinoxyfen^d^	Aug 2014	12	Inactive	4.79	1.21	10.1	> 75	Neg	2.5
Spiroxamine	Dec 2011	9	OD	Neg	—	9.29	—	Neg	NA
Ethoxyquin^e^	Mar 2008	8	AA	10.7	11.1	> 50	> 200	Neg	0.75

Insecticides									
Pirimiphos-methyl^d^	Sep 2017	30	OD	5.49	3.08	> 50	> 200	Neg	5.5
Endosulfan^e^	Jun 2006	19	AA	6.05	—	33.8	—	Neg	3.1
Methiocarb	Sep 2017	17	AA	6.82	—	> 46	—	Neg	2.5
Spirotetramat	Pending	17	OD	Neg	—	> 50	—	Neg	NA
Azinphos-methyl^d^,^e^	Jan 2007	16	OD	5.38	2.25	33.9	> 150	Neg	2.9
Bifenthrin^e^	May 2010	16	Active	Neg	99.8	22.2	> 200	Neg	NA
Indoxacarb	Mar 2016	16	OD	Neg	—	11.3	—	Neg	NA
Spinosad	Jan 2017	16	OD	Neg	—	13.1	—	Neg	NA
λ-Cyhalothrin^d^	Dec 2011	15	Inactive	23.1	95.4	51.4	> 200	Neg	0.65
Dicofol^e^	Mar 2009	15	AA	1.43	—	29.0	—	Neg	10.5
Bromopropylate^e^	Jul 2007	13	AA	0.540	—	27.2	—	Neg	24.1
Propargite^e^	Dec 2010	13	OD	Neg	—	0.487	—	Neg	NA
Fenitrothion^e^	Nov 2007	11	AA	0.098	—	> 50	—	4.9	112.2
Novaluron	Jul 2011	9	OD	Neg	—	> 50	—	Neg	NA
Profenofos^e^	Jul 2003	8	OD	Neg	—	8.61	—	Neg	NA
*p,p*′-DDE^e^	1986	—f	AA	0.948	—	> 50	—	3.6	NA

Herbicides									
Chlorpropham	Jan 2015	22	Inactive	7.66	10.2	> 50	> 40	2.67	2.9
Linuron	Dec 2013	12	AA	1.74	—	> 50	—	3.48	6.9

Abbreviations: AA, antiandrogenic, refers to known antiandrogens (not assessed by QSAR); EC_20_, concentration that produces a 20% effect; IC_20_, concentration that inhibits the androgenicity of DHT by 20%; NA, not applicable; Neg, no response was observed; OD, out of domain (QSAR was not able to predict activity for this compound).

a Expiration date is taken from Annex 1 of Council Directive 91/414/EEC concerning the “placing of plant protection products on the market” (European Union 1991).

b For details of exposure score, see text and Supplemental Material, Tables 1 and 2 (doi:10.1289/ehp.1002895).

c Androgenic in the absence of DHT.

d A newly described antiandrogenic compound.

e The expiration date is in the past, so the compound can no longer be used in Europe.

f Not included in ranked exposure.
